# Bridging the gap: Navigating the impact of dietary supplements on abdominal aortic aneurysm progression- A systematic review

**DOI:** 10.1371/journal.pone.0305265

**Published:** 2024-06-26

**Authors:** Zahra Amirsardari, Asal Khalili, Amir hossein Behnoush, Sadaf Agahi, Fatemeh Amirsardari, Erfan Kohansal, Parham Sadeghipour

**Affiliations:** 1 Cardiogenetic Research Center, Rajaie Cardiovascular Medical and Research Center, Iran University of Medical Sciences, Tehran, Iran; 2 School of Medicine, Tehran University of Medical Sciences, Tehran, Iran; 3 School of Nursing and Midwifery, Lorestan University of Medical Sciences, Lorestan, Iran; 4 Rajaie Cardiovascular Medical and Research Center, Iran University of Medical Sciences, Tehran, Iran; Army Medical University, CHINA

## Abstract

**Background:**

Vitamins D, E, A, B, C, and Omega-3 play crucial roles in modulating inflammatory and oxidative stress pathways, both implicated in abdominal aortic aneurysm (AAA) development. Recent research has explored the potential impact of dietary supplements on AAA progression. The systematic review aims to assess interventional studies investigating the effects of various dietary supplements on the development and severity of abdominal aortic aneurysms.

**Method:**

A systematic search using relevant keywords related to abdominal aortic aneurysm and dietary supplements was conducted across four databases (PubMed, Embase, Scopus, and Web of Science). Quality assessment for animal studies employed SYRCLE and the Cochrane Collaboration Risk of Bias Tool for randomized control trials. The study protocol is registered in PROSPERO under the registry code CRD42023455958.

**Results:**

Supplementation with Omega-3, Vitamins A, C, D, E, and the Vitamin B family exhibited positive effects in AAA progression. These supplements contributed to a reduction in AAA diameter, elastin degradation, inflammatory responses, and reactive oxygen species. Additional supplements such as Zinc, methionine, and phytoestrogen also played roles in mitigating AAA progression.

**Conclusion:**

The findings of this study underscore the potential role of dietary supplements in the progression of AAA. Predominantly based on animal studies, the results indicate that these supplements can limit AAA progression, primarily evidenced by their ability to mitigate inflammatory processes and oxidative stress pathways.

## 1. Introduction

The primary goal in the management of abdominal aortic aneurysm (AAA) is to prevent rupture, which is reported to have a mortality rate as high as 90 percent. The most critical determinant of rupture risk is the diameter of the aortic aneurysm [1], which is currently the primary criterion for intervention [2]. Large aneurysms (higher than 55 mm), are usually candidates for surgical/endovascular treatment, while smaller aneurysms are treated conservatively with a predefined imaging follow-up program [3]. Various non-invasive methods have been introduced to prevent aneurysm growth, but with controversial results [3].

The underlying pathogenesis of AAA involves aortic wall degeneration and aneurysmal formation, primarily encompassing chronic inflammation and oxidative stress [3]. These pathophysiological processes contribute to the activation of proteolytic and apoptotic signaling pathways, thereby initiating a gradual degradation of the extracellular matrix and cellular constituents that comprise the arterial wall [4]. Recently, several studies have suggested a potential correlation between serum levels of omega-3 fatty acids and vitamin D and the severity and prognosis of aneurysms [5–7]. Reduced oxidative stress and modulation of inflammatory process are proposed as potential mechanism of these supplements to prevent aneurysm progression. However, it is essential to note that the results of these studies remain highly contradictory, with some failing to observe any effect of dietary supplements on the progression of AAA [8–11].

Given that the management of most patients with aortic aneurysms involves surveillance, questions about the potential impact of dietary supplements on the progression of AAA have surged in recent years [12]. In this systematic review, our aim is to scrutinize all interventional studies, encompassing both pre-clinical and clinical studies, that have delved into the effects of various dietary supplements on the progression and severity of abdominal aortic aneurysms.

## 2. Methods

This systematic review has been conducted following the Preferred Reporting Items for Systematic Reviews and Meta-Analyses (PRISMA) guidelines [13]. The study protocol has also been registered in PROSPERO and is available at the following link: https://www.crd.york.ac.uk/prospero/display_record.php?ID=CRD42023455958.

### 2.1. Eligibility criteria

In this study, we included original human studies, comprising randomized controlled trials (RCTs) and prospective cohort studies, as well as animal model studies that have investigating the impact of dietary supplements on the development and progression of AAA. These supplements include the calciferol (vitamin D) family, vitamin A and retinoic acids, omega-3 and fish oil, the tocopherol (vitamin E) family, vitamin B family, vitamin C, and any other relevant supplements. We excluded studies that investigated other types of aneurysmal diseases, observational studies, as well as review studies, conference abstracts, and non-English studies.

### 2.2. Search strategy

A systematic search was conducted employing all pertinent keywords related to abdominal aortic aneurysm, dietary supplements, vitamin D family, vitamin A family, vitamin B family, omega-3, fish oil, vitamin C, and vitamin E, across the PubMed, Embase, Scopus, and Web of Science databases up until August 10th, 2023. This search encompassed studies published in the English language, and there were no restrictions regarding publication dates. Detailed search strategies can be found in S1 Table.

### 2.3. Selection process

**Fig 1:**
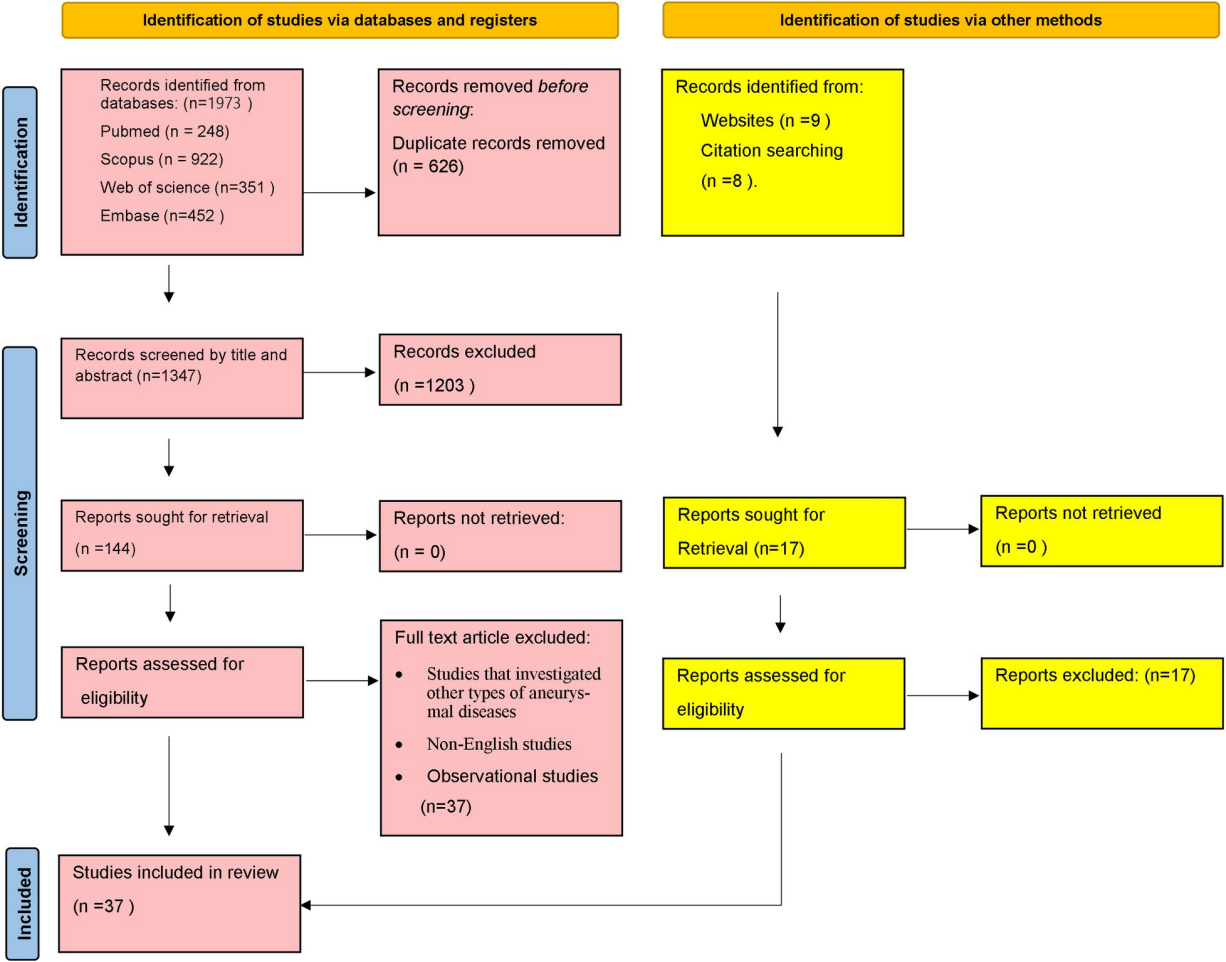
PRISMA flowchart of study selection and screening.

Two independent reviewers, ZA and AKH, initially screened the records by title and abstract using EndNote version 20 software. Subsequently, the studies underwent a full-text screening and were included in the systematic review. In the cases of disagreement between the two authors, a third author (AB) expertly intervened to resolve discrepancies and made the final decision. Fig 1 shows an overview of the entire selection process.

### 2.4. Data extraction

Two reviewers independently evaluated the quality of the publications and extracted pertinent information from the included studies. The extracted data encompassed details such as the first author’s name, publication year, country, characteristics and number of study subjects, study design, investigated supplements, their route of administration, and outcomes. These outcomes included changes in AAA incidence, rupture, diameter, and various pathophysiological changes such as inflammatory processes, oxidative stress, and extracellular matrix changes.

### 2.5. Risk of bias and study quality assessment

The quality of the animal studies was assessed using the SYstematic Review Centre for Laboratory animal Experimentation (SYRCLE) [14] and the Cochrane Collaboration Risk of Bias Tool for randomized control trials [15]. Two independent reviewers (ZA and AKH) conducted a comprehensive evaluation of the quality for each included study. Disagreements were resolved by a third reviewer (AB).

## 3. Results

### 3.1. Baseline characteristic of the included studies

This systematic review encompasses 37 peer-reviewed studies, comprising 34 animal model investigations, 2 randomized control trials, and 1 in vitro study on human tissues. Concerning animal models of AAA, the predominant model employed was Angiotensin II-induced AAA in mice, utilized in 18 studies followed by Elastase-induced (6 studies) [7, 16, 17], CaCl2-induced (5 studies), porcine pancreatic elastase (PPE)-induced (3 studies), hypoperfusion-induced (2 studies), nicotine-induced (1 study), and anti- Transforming growth factor-β (TGF-β)-induced (1 study). The two randomized control trials collectively encompassed a participant pool of 29,148 individuals. The most frequently explored supplements in the included studies were omega-3 fatty acids and their derivatives (13 studies), the vitamin B family (4 studies), the vitamin D family (3 studies), the vitamin A family (3 studies), methionine (3 studies), vitamin E (3 studies), and vitamin C (2 studies).

**Fig 2:**
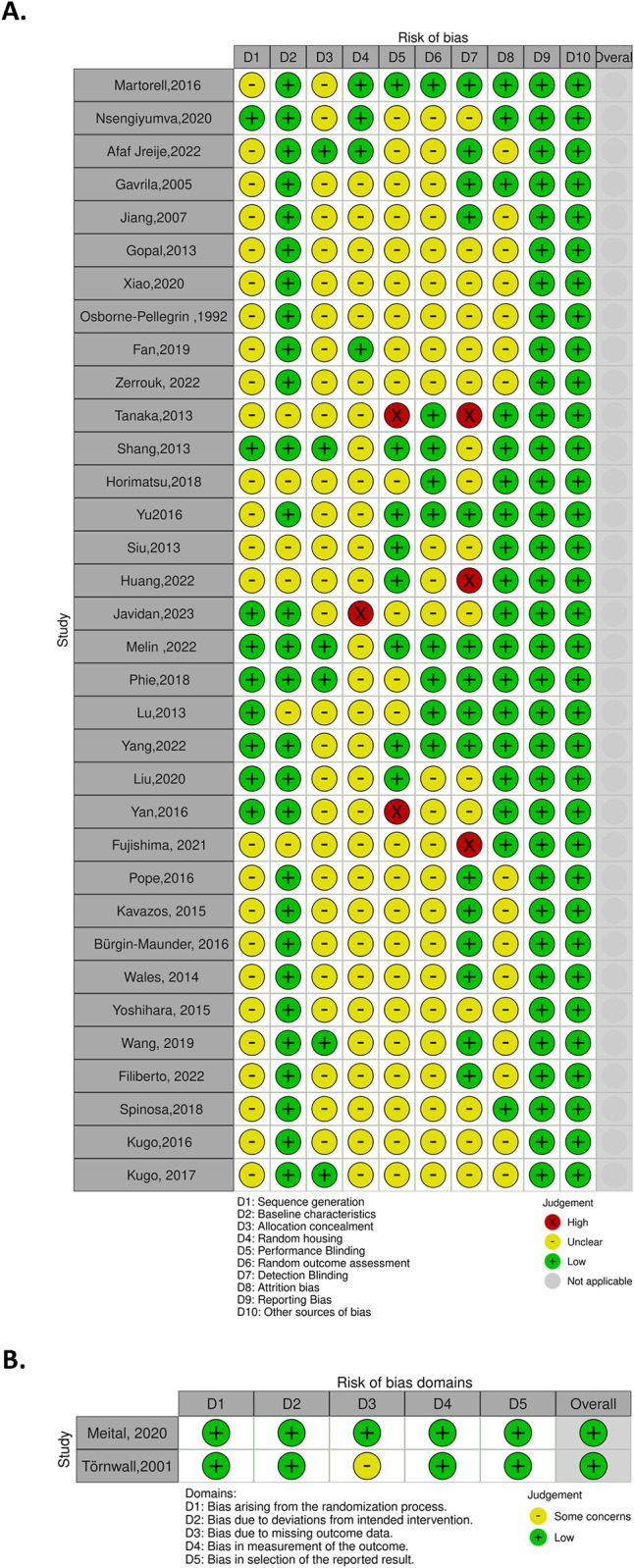
A) SYRCLE risk of bias assessment for in vivo studies, B) Cochrane Collaboration Risk Of Bias tool for randomized control trials.

Regarding the risk assessment of in vivo studies, the SYRCLE scores ranged from 3 to 9, with an average score of 5.38. As for the risk assessment of the two randomized control trials using the Cochrane Collaboration Risk of Bias tool, one demonstrated low risk across all domains of bias, while the other exhibited some issues related to missing outcome data bias and was low risk for other domains. A detailed risk of bias assessment is provided in Fig 2.

### 3.2. Omega-3 supplement

Thirteen studies [7, 8, 16–26] assessing the effect of omega-3 or its derivatives on AAA were included in this systematic review. These comprised one randomized control trial [18], one in vitro study using macrophage cells derived from patients with AAA [21], and eleven in vivo studies conducted on animal models of AAA [7, 8, 16, 17, 19, 20, 22–26].

#### 3.2.1. Animal studies

Dietary supplementation with n-3 PUFA resulted in a non-significant decrease in death due to abdominal aortic rupture, dissection, and elastin degradation [7, 16, 17]. This supplementation also led to a substantial decrease in MMP-9 immunoreactivity, neutrophil and macrophage infiltration, and superoxide production within the abdominal aorta walls, along with an increase in the expression of TGFβ-1 and tissue inhibitor of metalloproteinase 1 (TIMP-1) [7, 17].

The administration of Eicosapentaenoic acid (EPA) and docosahexaenoic acid (DHA) resulted in a decrease in AAA formation and aortic diameter. Other protective effects of EPA and DHA against AAA progression include reducing macrophage infiltration and the expression of inflammatory mediators such as chemoattractant protein-1, tumor necrosis factor-α, transforming growth factor-β, MMP-2, MMP-9, calcification factor Tnfsf11, and vascular cell adhesion molecule-1 in the aorta. Moreover, there was an observed shift in macrophage polarization towards an anti-inflammatory phenotype following the administration of EPA and DHA [20, 22].

Injection of RvD1 and RvD2 reduced aneurysmal diameter, elastin degradation, and macrophage infiltration. This treatment also decreased inflammatory cytokines, MMP-2 and MMP-9 activity, and NETosis biomarkers (including citrullinated histone H3 and neutrophil elastase levels). RvD2 injection increased anti-inflammatory cytokine levels and altered macrophage polarization towards an anti-inflammatory phenotype. RvD1 exhibited a capacity to mitigate AAA progression by reducing formyl peptide receptor 2 (FPR2), a member of the G-protein-coupled receptors (GPCRs) superfamily that plays crucial roles in the inflammatory process by interacting with various peptides and ligands [19, 23, 24].

Considering that EPA is primarily incorporated into phosphatidylcholine (PC) in the cell membrane, Fujishima et al. analyzed the distribution of EPA-containing PC in the AAA wall and its correlation with cellular and pathological changes in the AAA walls of fish oil-supplemented rats. Their findings revealed the accumulation of EPA-containing PC in a region of the aortic wall tissue associated with an increase in anti-inflammatory macrophages (M2) and a decrease in MMP-2 and malondialdehyde, both of which are oxidative stress markers [25].

Supplementation with fish oil, a rich source of EPA and DHA, significantly reduced aortic dilation diameter, aneurysmal rupture, and decreased levels of MMP-2, MMP-9, MMP-12, and mouse anti-malondialdehyde [8, 26].

#### 3.2.2. Human studies

A single study involving human subjects, which was a randomized controlled trial, has been included in this systematic review [18]. In a randomized double-blind controlled trial conducted by Meital et al. [18], 15 male AAA patients with a small AAA (< 5 cm), aged between 60 to 86, with a BMI < 39, and without cardiac arrhythmia, heart failure, symptomatic aortic stenosis, or chronic obstructive pulmonary disease were administered 1.5 g/day of DHA and 0.3 g/day of EPA for twelve weeks. This supplementation resulted in a significant reduction in pulse wave velocity (PWV), which indicates vascular stiffness, in patients with AAA compared to their baseline PWV. There was no observed change in the PWV of the control group.

#### 3.2.3. In vitro studies

Regarding in vitro studies, one study investigated the effect of n-3 PUFA, specifically DHA, on macrophages from AAA patients [21]. The study’s results indicated that DHA supplementation significantly lowered TNF-α and IL-6 levels, concurrently boosting glutathione peroxidase activity and heme oxygenase-1 mRNA expression in macrophages of AAA patients. These findings collectively indicate a notable reduction in oxidative stress within these cells. Table 1 summarizes the studies examining the impact of fish oil, n-3 PUFA, RvD1, and RvD2 on the pathogenesis and progression of AAA.

**Table 1:** Summary of fish oil, n-3 PUFA, RvD1, and RvD2 studies.

Supplement	Study	Country	Rout of administration	Sample	Study design*	Sample Size^a^	Age	Main Finding
Fish Oil	Kugo, 2016	Japan	Oral	male Sprague‐Dawley	in vivo- Hypoperfusion model	9	6 weeks	Reduced AAA diameter and mortality
								Decreased MMP-2, MMP-9, and mouse anti-malondialdehyde expression
				rats				
	Kugo, 2017	Japan	Oral	Male C57BL/6J mice	in vivo- Nicotine model	6	3 weeks	Decrease in elastin degradation, MMP-2 expression, Gelatinolytic activity and monocyte/macrophage infiltration
	Fujishima, 2021	Japan	Oral	male Sprague‐Dawley	in vivo- Hypoperfusion model	8	5 weeks	Increase in anti-inflammatory macrophages
								Decrease in MMP-2 and malondialdehyde expression
				rats				
N-3 PUFA	Wales, 2014	Australia	Oral	ApoE^−/−^ mice	in vivo- Ang-II model	20	3–4 weeks	Decrease in neutrophil and macrophage infiltration and superoxide production
								A slight decrease in dissection and death due to AAA rupture
	Kavazos, 2015	Australia	Oral	Male apoE^−/−^ mice	in vivo- Ang-II model	10	3 weeks	Decreased MMP-9 immunoreactivity
								Increased TIMP-1 and TGF-β1
	Yoshihara, 2015	Japan	Oral	Male apoE^−/−^ C57BL/6 mice	in vivo- Ang-II model	35		Reduced macrophage infiltration and inflammatory mediators expression
	Bürgin-Maunder, 2016	Australia	Oral	Male apoE^−/−^ mice	in vivo- Ang-II model	14	3–4 weeks	A modest extension in the time to death resulting from AAA rupture.
	Wang, 2014	Japan	Oral	Male BALB/cA mice	in vivo- CaCl_2_ model	13	7–9 weeks	Reduced aortic diameter and calcification, and elastin degradation
								Decrease in MMP-2, MMP-9 and RANKL expression
	Meital, 2019	Australia	Oral	AAA patients	in vitro	19	74.6±5.8 years	Reduced oxidative stress by lowering TNF-α and IL-6 levels
	Meital, 2020	Australia	Oral	AAA patients with small(<5cm) aneurysm	Randomized Controlled Trial	Omega3 group:15	Omega3 group: 73.6±5.0	Decrease in Pulse Wave velocity
						Placebo group: 15	Placebo group: 75.1 ± 5.7	
RvD1 and RvD2	Pope, 2016	USA	Intraperitoneal injection	Male C57BL/6J mice	in vivo- PPE and Ang-II models	18	8–12 weeks	Decrease in aortic diameter
								Reduced elastin degradation, macrophage infiltration and inflammatory cytokines
RvD1	Spinosa, 2017	USA	Intravenous injection	Male C57BL/6 mice and apoE^−/−^ mice	in vivo- topical lastase and Ang-II models	18	8–12 weeks	Reduced AAA diameter
	Filiberto, 2022	USA	Injection	Male C57BL/6 and FPR2^−/−^ mice and human AAA tissue	in vivo- PPE model	6–16	8–12 weeks	Reduced aneurysmal formation and inflammatory cytokines expression
					in vitro			

The abbreviations used in this table are as follows: AAA (Abdominal aortic aneurysm), RCT (Randomized controlled trial) MMP (Matrix Metalloproteinase), TIMP-1 (Tissue inhibitor of metalloproteinases-1), TGF-β (Transforming growth factor-β), IL-6 (Interleukin-6), and TNF-α (Tumor necrosis factor-α). *The study design in animal experiments encompasses various methods employed to induce abdominal aortic aneurysm (AAA) in mice. These approaches include the utilization of angiotensin-II (Ang-II), porcine pancreatic elastase (PPE), hypoperfusion, and CaCl2 to induce AAA. *Sample size in both animal and human studies encompasses intervention group.

### 3.3. Vitamin D

Three studies investigating the impact of the vitamin D family on AAA progression have been included in this systematic review [27–29]. All three studies utilized angiotensin-induced AAA mouse models. Two of these studies explored the effects of calcitriol [27, 28], while the remaining study investigated the influence of cholecalciferol on AAA in mouse models [29]. Notably, the latter study also examined the impact of the active vitamin D metabolite on human aortic smooth muscle cells in vitro. It’s important to note that our search did not yield any clinical studies concerning vitamin D in human subjects.

#### 3.3.1. Animal studies

In angiotensin II-induced AAA mice, vitamin D deficiency was associated with increased MMP-9 activity and elastin degradation [27]. Calcitriol supplementation reduced aortic aneurysm diameter, dissection, and rupture [27, 28]. This administration also reduced elastin degradation, macrophage infiltration, and expression of MMP-2, MMP-9, proinflammatory and proangiogenic chemokines (CCL2, CCL5, and CXCL1), vascular endothelial growth factor (VEGF) within the abdominal aortic walls. reduced expression of proinflammatory and proangiogenic chemokines resulted in decreased neovascularization, a risk factor for aneurysmal rupture. In addition, calcitriol supplementation increases the expression of tissue inhibitor of metalloproteinases-1 (TIMP-1), which is an endogenous inhibitor of MMP-9 and MMP-2. Moreover, calcitriol treatment led to a reduction in mitogen-activated protein kinases (MAPKs) and nuclear factor-κB (NF-κB) pathways, which are associated with oxidative stress [28]. Calcitriol supplementation by downregulating appotitic mediators(caspase-3, caspase-8, Bid, and t-Bid) and improvement of the Nrf2 pathway throughreducing the IFN-γ/IL-10 ratio mitigates apoptosis [27].

Along with calcitriol, a cholecalciferol-enriched diet resulted in a reduction in aneurysm diameter and a lower rupture rate of AAAs. human aortic muscle smooth cells exposed to the active metabolite of vitamin D, 1,25-dihydroxy vitamin D3, exhibited an upregulation of sclerostin expression. Moreover, there was an increase in the expression of contractile genes, including calponin 1 (CNN1), myosin heavy chain 11 (MYH11), smoothelin (SMTN), caldesmon (CALD1), transgelin (TAGLN), and α-SMA (ACTA2), all of which play a pivotal role in extracellular matrix remodeling (30).

Despite differences in study designs and outcome assessments, all preclinical studies consistently demonstrated the effectiveness of vitamin D supplementation in mitigating the progression of AAA.

### 3.4. Vitamin E

Four studies investigating the impact of Vitamin E on AAA were included based on the inclusion criteria [11, 30–32]. These comprised three animal studies [11, 30, 32] that employed angiotensin-induced AAA mouse models to evaluate the effects of vitamin E supplements. One of the studies was a randomized controlled trial that evaluated the impact of the active form of vitamin E, alpha-tocopherol, on a large population of male smokers [31].

#### 3.4.1. Animal studies

Gavrila et al. reported that four weeks of supplementation with a vitamin E-enriched diet significantly reduced AAA formation induced by angiotensin II infusion in apolipoprotein E-knockdown mice. This supplementation led to a 24% reduction in maximal aortic diameter, and a 44% reduction in the composite endpoint of fatal and nonfatal aortic rupture. Furthermore, vitamin E resulted in a decrease in oxidative stress, macrophage infiltration, and osteopontin expression within the aneurysmal tissues. However, the administration of vitamin E did not have a notable impact on aortic root atherosclerosis, MMP2 or MMP-9 activation, lipid profile, or systolic blood pressure [11].

In a subsequent study, Jiang et al. examined the protective effects of antioxidants on AAA formation in aged mice using the same mouse model of AAA. They supplemented mice with a combination of two antioxidants, vitamin E and vitamin C. While the combination of vitamin E and vitamin C slightly reduced the incidence of AAA rupture, it did not reach statistical significance [30].

In another study using an angiotensin II-infused mice model, Gopal et al. revealed that the administration of α-tocopherol resulted in a significant decrease in aortic diameter, circulating macrophages, and infiltrated macrophages. Additionally, decreased MMP-2, MMP-9, MMP-12, and uPAR mRNA levels, and increased ICAM-1 and VCAM-1 mRNA levels were observed in the α-tocopherol-treated group compared to the control group. [32].

#### 3.4.2. Human studies

In a randomized controlled trial involving a considerable number of male smokers, the potential preventive effects of a daily dose of 50 mg of alpha-tocopherol on the development of AAA were examined over a follow-up period of 5.8 years. In this RCT involving 29,133 male smokers, the participants were divided into four groups, each containing 7,286 individuals. One group received beta-carotene, another group received alpha-tocopherol, a third group received both beta-carotene and alpha-tocopherol, and the fourth group served as a control, receiving neither supplement. The study population comprised individuals aged 50–69, without prior cancer or any other serious disease. The findings of the study suggested that while alpha-tocopherol had a slight impact, it was not statistically significant in reducing the incidence of either ruptured or non-ruptured AAA [31].

### 3.5. Vitamin A

This review encompasses three studies that examine the impact of vitamin A or its derivatives on the progression of abdominal aortic aneurysms [31–33]. Among these studies, one involves human subjects [31], while the other two are animal studies utilizing angiotensin-induced AAA models [32, 33].

#### 3.5.1. Animal studies

In 2013, Gopal et al. evaluated whether β-carotene supplementation could reduce the severity or progression of abdominal aortic aneurysm in angiotensin-induced mouse models. The study found that administration of β-carotene resulted in a significant decrease in aortic diameter, compared to the control group. Additionally, animals treated with β-carotene exhibited a significant increase in LDL, cholesterol, and triglyceride levels, along with a decrease in HDL levels. Histopathological findings indicated a complete resolution of areas affected by atheromatous plaque in the aorta with evidence of disruption in the endothelial layer within the intima of the aorta. Furthermore, β-carotene utilization reduced the infiltration of inflammatory cells in the intima layer and restored circulating lymphocytes to normal levels [32].

Administration of all-trans-retinoic acid (ATRA) in aneurysmal mice resulted in a decrease in the abdominal aorta diameter compared to the control group. In the ATRA-treated group the expression of MMP 2, 9 and angiotensin II receptor type 1 were decreased. Furthermore this supplementation led to a reduction in elastin degradation [33].

#### 3.5.2. Human studies

In a randomized clinical trial investigated the potential preventive effects of β-carotene supplementation on the risk of developing large AAA among male smokers over a period of 5.8 years. The results indicated that β-carotene did not have a significant preventive effect on the risk of large AAA in this particular population (RR = 0.93, CI = 0.69–1.24) [31].

### 3.6. Vitamin C

This systematic review included three studies investigating the effect of vitamin C on the progression of AAA. Unfortunately, we were unable to find any human clinical trials involving vitamin C.

The initial study investigated the effects of ascorbic acid on a rat model of saccular abdominal aortic aneurysm. In the treatment group, the aorta was wrapped with a gelatin hydrogel sheet incorporating ascorbic acid. Treatment with ascorbic acid resulted in the preservation of elastin and collagen, the downregulation of inflammatory markers (MMP-9, IL-1β, TNF-α, and monocyte chemotactic protein-1), and the upregulation of tissue inhibitors of metalloproteinase. Additionally, the treated group exhibited lower levels of reactive oxygen species and fewer CD68-positive cells [34].

Another study investigated the effects of Vitamin C administered through intraperitoneal injections. The treated group showed a reduced aneurysmal diameter, enhanced preservation of elastin content, and decreased levels of oxidative stress markers. Additionally, in the treated group, there was a significant reduction in the expression of TNF-α, MMP-2, MMP-9, IL-1β, and IL-6. However, there was an increase in the expression of tissue inhibitors of metalloproteinases [35].

In a study by Jiang et al., mentioned earlier in the vitamin E section, supplementation was examined in a mouse model of AAA using a combination of orally administered vitamin E and vitamin C. They demonstrated a slight reduction in the incidence of AAA rupture, although it did not reach statistical significance [30].

### 3.7. Vitamin B family

This systematic review encompasses four animal studies that investigated the impact of the vitamin B family on AAA progression. These studies utilized CaCl2- or angiotensin-induced AAA models [9, 36–38]. Among them, two studies examined the impact of vitamin B9 [37, 38], one focused on riboflavin [36], and another investigated niacin [9]

#### 3.7.1. Animal studies

Supplementation with niacin reduced aorta diameter and prevented aneurysmal formation. It resulted in decreased macrophage infiltration, mitigated elastin degradation, and lowered the expression of MMP-2, MMP-9, MCP-1, and TNF-α. Importantly, the inhibitory effects of niacin against AAA formation were found to be independent of GPR109A [9].

The administration of riboflavin resulted in a significant increase in the activity of a critical antioxidant enzyme, superoxide dismutase, while simultaneously reducing ROS levels, 8-OHdG-positive and CD68-positive cells, as well as the expression of MMP-9 and TNF-α. Furthermore, the treated group exhibited reduced aortic dilation, less elastin degradation, and decreased inflammatory cell infiltration compared to the control group [36].

Oral folic acid treatment significantly decreased AAA formation, elastin degradation and macrophage infiltration. It also decreased superoxide production and increased H4B bioavailability, a cofactor of eNOS, promoting the recoupling of eNOS and leading to higher NO levels [37]. In another study, the potential benefits of combining folic acid with Nifedipine were investigated. The study revealed that oral administration of folic acid plus Nifedipine significantly reduced AAA incidence. The combination therapy effectively decreased aortic superoxide production, eNOS uncoupling activity, elastin degradation, and adventitial hypertrophy [38].

### 3.8. Methionine

Red meat constitutes a significant dietary source of methionine, a precursor for homocysteine and cysteine. Elevated intake of methionine and subsequent homocysteine levels have been linked to an increased risk of cardiovascular disease, primarily attributed to the induction of oxidative stress and inflammatory cascades. Methionine-induced hyperhomocysteinemia has been associated with various alterations in the structure of the aortic wall, including thickening of layers, endothelial cell hyperplasia, microthrombi formation, atheromatous plaque development, collagen accumulation, and an increase in interlamellar spaces [39]. Observational studies have also indicated a potential association between hyperhomocysteinemia and the progression of AAA [40].

While one animal study explored the impact of methionine administration on aortic aneurysm [41], it is noteworthy that no human studies have been identified investigating methionine in this context.

In a study examining the effect of hypermethioninemia on AAA development in rats, findings revealed that hypermethioninemia exacerbated AAA severity and elastin degradation. Furthermore, hypermethioninemia was associated with increased expression of MMP-2, osteopontin, interleukin-6, and autophagy markers (beclin-1 and LC3) within aortic tissues [41].

### 3.9. Other supplements

Limited animal model studies have been conducted to investigate the effect of other supplemention on the AAA progression.

#### 3.9.1. Celastrol supplementation

In a mouse model of Ang II-induced Abdominal Aortic Aneurysm (AAA), dietary supplementation with Celastrol, a pentacyclic triterpene derived from the root extracts of the Chinese herb Tripterygium wilfordii, resulted in increased abdominal aortic dilatation, elastin degradation, and activation of MMP-9. [42].

#### 3.9.2. Cycloastragenol

In the investigation of cycloastragenol, the active compound found in the Chinese herb Astragalus membranaceus, and its effect on aneurysm progression in a rat model of elastase-induced AAA, significant findings emerged. The study revealed that in the group treated with cycloastragenol, there were notable reductions observed in aneurysmal diameter, microcalcification, MMP-2 activity, and elastin degradation. Additionally, levels of messenger RNA for various anti-oxidative and inflammatory markers (LOX, MMP-2, MMP-9, MMP-12, CD45, CD68, F4/80, iNOS, IL-10, IL-6, Nrf2, HO-1) remained unchanged [43].

#### 3.9.3. Tree nuts

A diet enriched with tree nuts did not yield a significant effect on the diameter of the aorta or the incidence of aortic rupture in mice [44].

#### 3.9.4. Dietary phytoestrogens

A diet rich in phytoestrogens was found to reduce the incidence of AAA and mitigate elastin and collagen degradation in male mice, but this effect was not observed in female mice. Within the male treatment group, significant reductions were noted in aortic neutrophil, macrophage, and lymphocyte counts. Moreover, levels of MMP-9, IL-1β, IL-6, IL-17, IL-23, MCP-1, RANTES (regulated on activation, normal T cells expressed and secreted), interferon-gamma, and TNF-α exhibited similar decreases in the aortas of treated males [45].

#### 3.9.5. Short-chain fatty acids

Oral administration of short-chain fatty acids (SCFAs), an intestinal microbial metabolite, resulted in a significant reduction in the maximum outer diameter of aneurysms in mouse models induced by elastase and Ca_3_(PO)_4_. Additionally, the mice treated with SCFAs exhibited a statistically significant decrease in medial elastin degradation and a reduced presence of CD3^+^ T cells and CD68^+^ macrophages within the aortic wall compared to the control groups [46].

#### 3.9.6. Spermidine

In a murine model of AAA, the administration of spermidine, a natural polyamine, demonstrated notable reductions in aneurysmal incidence, aortic diameter, neo-angiogenesis, as well as diminished collagen and elastin degradation in comparison to the control group. Spermidine treatment also led to diminished infiltration of macrophages, T cells, and neutrophils within the aorta. Moreover, it was observed that spermidine administration resulted in a decrease in the circulating inflammatory monocytes and neutrophils. Further investigation revealed alterations in autophagy within AAAs following spermidine treatment, potentially mediated through the mTOR and Beclin1 pathways [47].

#### 3.9.7. Zinc

Intraperitoneal administration of zinc in a rat model of AAA induced by CaCl2 led to a decrease in aneurysmal incidence, aortic diameter, and elastin fragmentation. Furthermore, zinc injection resulted in a reduction in the expression of MMP-2 and MMP-9. Additionally, zinc treatment decreased macrophage infiltration and suppressed the activation of NF-κB [48].

Table 2 summarizes the studies examining the impact of vitamin D, E, A, B, C families, and other supplements on the pathogenesis and progression of AAA.

**Table 2:** Summary of vitamin D, E, A, B, C families, and other supplementation studies.

Supplement	Study	Country	Rout of administration	Sample	Study Design	Sample Size*	Age	Main Finding
Vitamin D	Martorell, 2016	Spain	Oral- Calcitriol	Male, apoE^−/−^, BALB/c mice	in vivo- Ang-II model	24	4 weeks	Reduction in the formation and diameter of dissecting aneurysms.
	Nsengiyumva, 2020	Australia	Oral- Cholecalciferol	Male apoE^−/−^, C57BL/6J mice	in vivo- Ang-II model	41	13 weeks	Decrease in aneurysm diameter and rupture rate.
	Jreije, 2022	Lebonan	Intraperitoneal- Calcitriol	Male, BALB/c mice	in vivo- Ang-II and	10	4 weeks	Decrease in elastin degradation, incidence, and severity of AAA.
					anti-TGF-β models			
Vitamin E	Torrnwall, 2001	Finland	Oral- α-Tocopherol, β-Carotene	Smoker men	RCT	α-Tocopherol group: 7286	50–69 years	Slight, non-significant reduction in the incidence of AAA among individuals receiving α-tocopherol compared with those who did not (RR = 0.83)
						β-Carotene group: 7282		
						α-Tocopherol + β-Carotene group: 7278		
	Gavrila, 2005	USA	Oral- Vitamin E	Male apoE^−/−^ mice	in vivo- Ang-II model	4	6 months	Reduction in AAA formation, aortic diameter, and rupture.
	Jiang, 2007	Australia	Oral- Vitamin E plus Vitamin C	Male apoE^−/−^ mice	in vivo- Ang-II model	13	50–60 weeks	Slight, non-significant reduction in aneurysmal rupture.
	Gopal, 2013	India	Oral- α-Tocopherol	Male apoE^−/−^ mice	in vivo- Ang-II model	6	4 months	Decrease in aortic diameter.
Vitamin A	Torrnwall, 2001	Finland	Oral- α-Tocopherol, β-Carotene	Human	RCT	α-Tocopherol group: 7286	50–69 y	Slight, non-significant reduction in the incidence of AAA among individuals receiving β-Carotene compared with those who did not (RR = 0.93)
						β-Carotene group: 7282 α-Tocopherol + β-Carotene group: 7278		
	Gopal, 2013	India	Oral- β-Carotene	Male apoE^−/−^ mice	in vivo- Ang-II model	6	4 months	Decrease in aortic diameter.
	Xiao, 2020	China	Oral- all-trans retinoic acid	Male apoE^−/−^ mice	in vivo- Ang-II model	12	16 weeks	Decrease in aortic diameter.
Methionine	Fan, 2019	China	Intragastric- Methionine	Sprague-Dawley rats	in vivo- PPE model	15	8 weeks	Increase in aortic dilation.
Vitamin C	Tanaka, 2013	Japan	Aorta wrapped with a ascorbic acid hydrogel sheet	Male	in vivo- PPE and CaCl_2_ model	18	8 weeks	Reduction in elastin and collagen degradation.
				Sprague-Dawley rats				Decrease in reactive oxygen species levels.
	Shang, 2013	China	Intraperitoneal-Vitamin C	Male rats	in vivo- PPE model	12	6 weeks	Decrease in aortic diameter and oxidative stress markers.
Vitamin B	Siu, 2013	USA	Oral-Folic acid	Male apoE^−/−^ mice	in vivo- Ang-II model	41	6–8 months	Decrease in AAA incidence and aortic diameter.
	Yu, 2015	Japan	Gastric gavage-Riboflavin	Male Sprague-Dawley	in vivo- CaCl_2_ model	18	10 weeks	Decrease in aortic diameter and reactive oxygen species levels.
				rats				
	Horimatsu, 2019	USA	Oral- Niacin	Male Ldlr^−/−^ mice	in vivo- Ang-II and CaCl_2_ models			Decrease in macrophages infiltration and elastin degradation.
	Huang, 2022	USA	Oral- Folic acid	Male apoE^−/−^ mice	in vivo- Ang-II model	38	6–8 months	Decrease in AAA incidence.
Others	Lu, 2013	USA	Oral-Phytoestrogen	Female and male C57BL/6 mice	in vivo- PPE model	16	6 weeks	Decrease in aortic diameter and MMP-9 levels in male mice.
	Yan, 2016	China	Intraperitoneal- Zinc	Wistar rats	in vivo- CaCl2 model	10	8 weeks	Decrease in aortic diameter and inflammatory mediators.
	Phie, 2018	Australia	Oral- Tree nuts	Male apoE^−/−^ mice	in vivo- Ang-II model	17	10–12 weeks	No impact on aortic diameter or rupture.
	Liu, 2020	China	Oral- Spermidine	Male C57BL/6 mice	in vivo- PPE model	18	8–10 weeks	Decrease in aortic diameter.
	Yang, 2022	China	Oral-Short chain fatty acids	Male C57BL/6J mice	in vivo- PPE and Ca_3_(PO4)_2_ models	13	7–8 weeks	Reduction in aortic diameter, along with elastin degradation and macrophage infiltration.
	Melin, 2022	Denmark	Oral- Cycloastragenol	Male Sprague-Dawley rats	in vivo- PPE model	12	7–10 weeks	Reduction in aortic diameter and elastin degradation.
	Javidan, 2023	USA	Oral- Celastrol	Female and male Ldlr^−/−^ mice	in vivo- Ang-II model	24	8–12 weeks	Increase in aortic dilatation, AAA incidence, and elastin degradation.

The abbreviations used in this table are as follows: AAA (Abdominal aortic aneurysm), RCT (Randomized controlled trial). *The study design in animal experiments encompasses various methods employed to induce abdominal aortic aneurysm (AAA) in mice. These approaches include the utilization of angiotensin-II (Ang-II), porcine pancreatic elastase (PPE), hypoperfusion, CaCl_2,_ Ca_3_(PO4)_2_, and nicotine to induce AAA. *Sample size in both animal and human studies encompasses intervention group.

## 4. Discussion

To our knowledge, this is the first systematic review assessing the role of vitamin D, E, and omega-3 fatty acids in AAA. Our study incorporated human and animal studies in addition to in vitro studies in order to provide better insight into the topic. The findings of these studies can be summarized as following: 1) Although omega-3 supplementation did not result in significantly lower mortality from AAA in animal studies, it reduced aortic diameter significantly. Additionally, it reduced AAA incidence in murine models. In a human study, it decreased PWV representing lower vascular stiffness. 2) Vitamin D supplementation resulted in slower progression of AAA through reduction in diameter and a lower rupture rate in animal models. 3) Supplementation with vitamin E reduced AAA formation and aortic diameter in some mouse models. Moreover, it led to a non-significant reduction in the incidence of AAA in a human RCT. 4) Regarding vitamin A supplementation, despite the fact that in a study on angiotensin-induced mice, β-carotene decreased aortic diameter, β-carotene could not reduce the development of large AAA in an RCT. 5) Vitamin C supplementation resulted in a reduction of maximal aortic diameter. 6) Vitamin B family, particularly niacin, riboflavin, and folic acid, had positive effects on decreasing AAA diameter in mouse models.

It has been suggested that long-chain n-3 PUFAs are associated with a lower risk of mortality from inflammatory diseases [49]. To support this idea, AAA incidence has been suggested to be lower in the Japanese population, where dietary intake of EPA and DHA is fourfold higher than that reported in European cohorts [50, 51]. Although there might be confounding factors such as lower smoking rates in Japanese individuals, there is still a high possibility of n-3 PUFA involvement. Moreover, supplementation with n-3 PUFAs has shown to decrease levels of C-reactive protein, IL-6, and TNF-α, along with reducing the infiltration of macrophages and neutrophils into the aortic wall [52, 53]. Another way n-3 PUFAs could impact AAA progressions by elevating serum high-density lipoprotein (HDL) levels while reducing triglycerides and low-density lipoprotein (LDL), which are important predictors of AAA development [54, 55]. It appears that PUFAs are one of the most extensively researched supplements regarding their impact on AAA.

Given the roles of vitamin D in affecting the established risk factors of AAA such as hypertension, inflammation, and vascular calcification, it has been suggested that serum vitamin D may have a potential association with AAA development [56–59]. In a study conducted among older men, it was shown that the lowest quartile of serum 25(OH) vitamin D levels had a five-fold chance of AAA (>40 mm) compared with the upper quartile [5]. Another potential explanation for the association between vitamin D and AAA could be attributed to the involvement of bone proteins in AAA pathogenesis [60, 61]. For instance, research indicates a reduction in the expression of the bone protein sclerostin (SOST) in the aorta of individuals with AAA [61], while vitamin D has been observed to up-regulate SOST expression [62]. These findings suggest that vitamin D could be a promising candidate for larger randomized trials in patients with AAA.

Human AAA tissues also had an increased oxidative stress [63, 64]. Another interesting observation was made by Sakalihasan et al, who reported lower plasma vitamin E levels in patients with AAA but not in those with coronary artery disease, highlighting its specificity for AAA [65]. Oxidative stress is considered one of the main consequences of inflammation, which has been shown to be involved in AAA pathogenesis [66, 67]. In the study by Miller et al., it was demonstrated that aneurysmal aorta in humans has higher levels of oxidative damage and pro-oxidant enzyme activity [63]. Therefore, vitamin E, as a dietary anti-oxidant might be beneficial in patients with AAA. Similarly, Nakahashi et al. found that vitamin E supplementation could lead to a reduction in aortic enlargement and reactive oxygen species (ROS) production [68]. The same story could be said for β-carotene, which demonstrated efficacy in our systematic review. β-carotene may contribute to AAA progression by reducing inflammation severity and enhancing the immune system [69]. In summary, our findings suggest that vitamin E could be a potential candidate for further investigation in AAA.

The studies assessing hyperhomocysteinemia in relation to AAA progression vary based on population, definitions, and adjustment for confounding variables [70, 71]. The relationship between hyperhomocysteinemia and vascular diseases has been established and has become an interesting topic for research [72, 73]. One of the main causes of hyperhomocysteinemia is deficiencies in its cofactors, such as folate, vitamin B6, and vitamin B12 [74], and this could be the clue for the efficacy of the vitamin B group in AAA. However, as there were not many studies found for our systematic review, larger studies are warranted to exclusively assess vitamin B supplementation in human subjects and with a high sample size.

In addition to the main supplements assessed in our study, others such as methionine, celastrol, cycloastragenol, phytoestrogens, SCFAs, spermidine, and zinc were also assessed. Compared to vitamins, there were fewer studies for these supplements, and more research is necessary to confirm the preliminary findings.

This study was the most comprehensive systematic review for the assessment of dietary supplements in AAA. The inclusion of animal, in vitro, and human studies stands out as one of its main strengths. However, there are certain limitations that should be aknowleded. First, the limited number of studies included for each supplement underscores the need for larger future studies, particularly focusing on RCTs. Second, variations in study designs, settings, and populations among the included studies may restrict the generalizability of our findings. Third, due to the variety in the statistical methods among the included studies, we were not able to perform a meta-analysis and the lack of quantitative review in our study is another limitation. Finally, as the majority of our included studies were animal studies, there is a need for larger human studies.

## 5. Conclusion

The findings of these studies suggest that dietary supplements may play a role in the progression of AAA. The included studies primarily involved animals, indicating that these supplements could potentially limit AAA progression by reducing aortic diameter. Notably, among the investigated supplements, omega-3, vitamin E, and vitamin A demonstrated some effectiveness in human studies. Additionally, supplements such as vitamin C, the vitamin B family, and zinc showed promising results in animal studies but have yet to be evaluated in human studies, suggesting they merit consideration for future research. Finally, the precise efficacy of dietary supplements in limiting AAA progression and reducing mortality warrants additional evaluation, as current research has not yielded definitive conclusions.

## Supporting information

S1 Table Detailed search queries. (DOCX)

S1 File PRISMA checklist. (DOCX)

## Abbreviations

AAA: Abdominal aorta aneurysm
RCT: Randomized controlled clinical trial
PPE: Porcine pancreatic elastase
TGF-β: Transforming growth factor-β
n-3 PUFA: Omega-3 polyunsaturated fatty acids
MMP: Matrix metalloproteinase
TIMP-1: Tissue inhibitor of metalloproteinase 1
EPA: Eicosapentaenoic acid
DHA: Docosahexaenoic acid
Tnfsf11: Tumor necrosis factor ligand superfamily member 11
RvD1/2: Resolvin D1/D2
NETosis: A program for formation of neutrophil extracellular traps
FPR2: Formyl peptide receptor 2
GPCRs: G-protein-coupled receptors
PC: Phosphatidylcholine
PWV: Pulse wave velocity
TNF-α: Tumor necrosis factor alpha
IL: Interleukin
Ang-II: Angiotensin-II
CCL: C–C motif chemokine ligand
CXCL: C-X-C motif chemokine ligand
VEGF: Vascular endothelial growth factor
MAPK: Mitogen-activated protein kinase
NF-κB: Nuclear factor-κB
BiD: BH3 Interacting Domain Death Agonist
t-BiD: truncated p15 BID
Nrf2: The nuclear factor erythroid 2–related factor
IFN-γ: Interferon gamma
CNN1: Calponin 1
MYH11: Myosin heavy chain 11
SMTN: Smoothelin
CALD1: Caldesmon1
TAGLN: Transgelin
ACTA2: α-SMA
α-SMA: alpha-smooth muscle actin
ATRA: All-trans-retinoic acid
MCP: Monocyte chemotactic protein
ROS: Reactive oxygen species
8-OHdG: 8-Hydroxy-2’-deoxyguanosine
eNOS: endothelial nitric oxide synthase
H4B: Tetrahydrobiopterin
LC3: Light chain 3
CAG: Cycloastragenol
LOX: Lysyl oxidase
HO-1: Heme oxygenase 1
RANTES: Regulated upon Activation, Normal T Cell Expressed and Presumably Secreted
SCFA: Short-chain fatty acids
mTOR: Mammalian target of rapamycin
HDL: High density lipoprotein
LDL: Low density lipoprotein
CRP: C- reactive protein
SOST: Sclerostin
