# Supplementary material for: Bridging the gap: Navigating the impact of dietary supplements on abdominal aortic aneurysm progression- A systematic review
Source: PLoS One. 2024 Jun 26;19(6):e0305265. doi: 10.1371/journal.pone.0305265 (PMC11207180; doi:10.1371/journal.pone.0305265)
Supplement: S1 Table — (DOCX) [file pone.0305265.s001.docx]

Supplementary Table 1- Detailed search queries.

| Pubmed | #1 | "Aortic Aneurysm, Abdominal"[Mesh] OR [Abdominal Aortic Aneurysms] OR [Aneurysm, Abdominal Aortic] OR [Abdominal Aortic Aneurysm] OR [Abdominal Aorta Aneurysm] OR [Abdominal Aorta Aneurysms] OR [Aorta Aneurysm, Abdominal] OR [Aneurysm, Abdominal Aorta] OR "Aortic Aneurysm, Thoracoabdominal"[Mesh] OR [Aneurysm, Thoracoabdominal Aortic] OR [Thoracoabdominal Aortic Aneurysms] OR [Thoracoabdominal Aortic Aneurysm] OR [TAA Thoracoabdominal Aortic Aneurysm] |
| --- | --- | --- |
|  | #2 | ((((("Vitamin D"[Mesh] OR "Cholecalciferol"[Mesh] OR "Hydroxycholecalciferols"[Mesh] OR "Calcifediol"[Mesh] OR "Dihydroxycholecalciferols"[Mesh] OR "Calcitriol"[Mesh] OR "Ascorbic Acid"[Mesh] OR [Dihydroxyvitamins D] OR [Vitamin D3] OR [Cholecalciferols] OR[Calciol]OR[Vitamin D 3]OR[Vitamin D3]OR [Hydroxyvitamins D] OR[Hydroxycholecalciferol]OR [25-Hydroxyvitamin D 3]OR[25 Hydroxyvitamin D 3]OR[25-Hydroxyvitamin D3]OR[25 Hydroxyvitamin D3]OR[25-Hydroxycholecalciferol] OR[1 alpha,25-Dihydroxycholecalciferol]OR[1,25-Dihydroxycholecalciferol]OR[1,25 Dihydroxyvitamin D3] OR [Calcitriol]) OR ("Vitamin E"[Mesh] OR "Tocopherols"[Mesh] OR "alpha-Tocopherol"[Mesh] OR "beta-Tocopherol"[Mesh] OR "gamma-Tocopherol"[Mesh] OR [Tocopherol] OR[Vita E] OR [VitaE] OR[Uno Vit] OR [Tocopherol Acetate] OR [Tocopheryl] OR [beta Tocopherol] OR [gamma-Tocopherol])) OR ("Carotenoids"[Mesh] OR "Retinoids"[Mesh] OR "Vitamin A"[Mesh] OR "Tretinoin"[Mesh]) OR "Alitretinoin"[Mesh] OR [Carotenoid] OR [Carotenes] OR [Carotene] OR [Retinoid] OR [All-Trans-Retinol] OR [All Trans Retinol]OR [Retinol]OR [11-cis-Retinol] OR[Retinoic Acid]OR[Acid, Retinoic]OR[Vitamin A Acid]OR[all-trans-Retinoic Acid]OR[all trans Retinoic Acid]OR[trans-Retinoic Acid]OR[trans-Retinoic]OR[trans Retinoic Acid]OR[Retin-A]OR[Retin A]OR[beta-all-trans-Retinoic Acid]OR[all trans Retinoic Acid]OR[Tretinoin])) OR ("Fatty Acids, Omega-3"[Mesh] OR [Omega-3 ]OR[Omega3]OR[Omega 3]OR[n-3 Oil]OR[Oil, n-3]OR[n 3 Oil]OR[n3 Oil]OR[Oil, n3]OR[n-3 Fatty Acids]OR[n 3 Fatty Acids]OR[ Omega 3 Fatty Acids]OR[n-3 PUFA]OR[PUFA, n-3]OR[n 3 PUFA]OR[n3 Fatty Acid]OR[Fatty Acid, n3]OR[n3 PUFA]OR[PUFA, n3]OR[n3 Polyunsaturated Fatty Acid]OR[n3 Oils]OR[n-3 Oils]OR[n 3 Oils]OR[N-3 Fatty Acid]OR[Acid, N-3 Fatty]OR[Fatty Acid, N-3]OR[N 3 Fatty Acid]OR[n-3 Polyunsaturated Fatty Acid]OR[n 3 Polyunsaturated Fatty Acid])) OR ("Dietary Supplements"[Mesh]OR[Supplement]OR[Supplements, Dietary]OR[Dietary Supplementations]OR[Supplementations, Dietary]OR[Food Supplementations]OR[Food Supplements]OR[Food Supplement]OR[Supplement, Food]OR[Nutraceuticals]OR[Nutraceutical]OR[Nutriceuticals]OR[Nutriceutical]OR[Neutraceuticals]OR[Neutraceutical]OR[Supplements])) OR ("Fish Oils"[Mesh]OR[Oils, Fish]OR[Fish Oil]OR[Oil, Fish]OR[Fish Liver Oils]OR[Liver Oils, Fish]OR[Oils, Fish Liver] OR [Ascorbic Acid] OR [Vitamin C] OR [Ascorbate] OR "Ascorbic Acid"[Mesh] OR [Vitamin C] OR [Ascorbate] OR "Vitamin B 6"[Mesh] OR [Pyridoxal] OR [Pyridoxin] OR [Pyridoxol] OR [Vitamin B 6] OR [Vitamin B6] OR [Vitamin B 2] OR [Vitamin B2] OR [Vitamin B 12] OR [Vitamin B12] OR [Vitamin B] OR [Riboflavin] OR "Riboflavin"[Mesh] OR "Vitamin B 12"[Mesh] OR [Cobalamin] OR [Cobalamin]) |
|  | final | (#1) AND (#2) |
| Embase | 1 | "Aortic Aneurysm*" OR "Abdominal Aortic Aneurysm*" OR "Abdominal Aorta Aneurysm*" OR "Thoracoabdominal Aortic Aneurysm*" |
|  | 2 | "Vitamin D3" OR "Vitamin D*" OR " Cholecalciferol*" OR " Hydroxycholecalciferol*" OR "Hydroxyvitamins D* " OR "Calcifediol" OR "25-Hydroxyvitamin D 3" OR " 25-Hydroxyvitamin D* " OR "25 Hydroxyvitamin D 3" OR " 25 Hydroxyvitamin D*" OR " 25-Hydroxycholecalciferol" OR "25 Hydroxycholecalciferol*" OR "25 Hydroxycholecalciferol*" OR "Dihydroxycholecalciferol*" OR "Dihydroxyvitamins D" OR "Calcitriol" OR "1,25-Dihydroxycholecalciferol" OR "1,25 Dihydroxycholecalciferol" OR "1,25-Dihydroxyvitamin D3" OR "1,25 Dihydroxyvitamin D3" OR "Vitamin A " OR "Retinol" OR " All Trans Retinol" OR " cis Retinol " OR "Tretinoin " OR "Retinoic Acid" OR " Acid Retinoic " OR "all-trans-Retinoic Acid " OR "all trans Retinoic Acid" OR " trans-Retinoic Acid " OR "trans Retinoic Acid" OR " Retin A" OR " Alitretinoin" OR " 9-cis-Retinoic Acid" OR " cis Retinoic Acid" OR "Vitamin E" OR "Tocopherol*" OR "Tocovital" OR "VitaE" OR "Uno Vit" OR "alpha-Tocopherol" OR "alpha Tocopherol" OR "beta-Tocopherol" OR "gamma-Tocopherol" OR "Omega-3 " OR " Omega 3" OR " Omega3 " OR "n-3 Oil " OR "n 3 PUFA" OR " n3 Fatty Acid " OR "n3 Polyunsaturated Fatty Acid" OR " n3 Oil*" OR " n 3 Polyunsaturated Fatty Acid" OR "alpha Linolenic Acid" OR "alpha-Linolenic Acid" OR "Linolenic Acid" OR "Docosahexenoic Acid*" OR "Docosahexaenoate" OR "Eicosapentaenoic Acid" OR "Dietary Supplement* " OR "Nutraceutical* " OR "Fish Oil* " OR "Fish Liver Oil*" OR "Ascorbic Acid" OR "Vitamin C" OR "Ascorbate" OR "Vitamin B 6" OR "Pyridoxal" OR "Pyridoxin" OR "Pyridoxol" OR "Vitamin B 6" OR "Vitamin B6" OR "Vitamin B 2" OR "Vitamin B2" OR "Vitamin B 12" OR "Vitamin B12" OR "Vitamin B" OR "Riboflavin" OR "Riboflavin" OR "Vitamin B 12" OR "Cobalamins" OR "Cobalamin" |
|  | final | 1 and 2 |
| scopus | 1 | "Aortic Aneurysm*" OR "Abdominal Aortic Aneurysm*" OR "Abdominal Aorta Aneurysm*" OR "Thoracoabdominal Aortic Aneurysm*" |
|  | 2 | "Vitamin D3" OR "Vitamin D*" OR " Cholecalciferol*" OR " Hydroxycholecalciferol*" OR "Hydroxyvitamins D* " OR "Calcifediol" OR "25-Hydroxyvitamin D 3" OR " 25-Hydroxyvitamin D* " OR "25 Hydroxyvitamin D 3" OR " 25 Hydroxyvitamin D*" OR " 25-Hydroxycholecalciferol" OR "25 Hydroxycholecalciferol*" OR "25 Hydroxycholecalciferol*" OR "Dihydroxycholecalciferol*" OR "Dihydroxyvitamins D" OR "Calcitriol" OR "1,25-Dihydroxycholecalciferol" OR "1,25 Dihydroxycholecalciferol" OR "1,25-Dihydroxyvitamin D3" OR "1,25 Dihydroxyvitamin D3" OR "Vitamin A " OR "Retinol" OR " All Trans Retinol" OR " cis Retinol " OR "Tretinoin " OR "Retinoic Acid" OR " Acid Retinoic " OR "all-trans-Retinoic Acid " OR "all trans Retinoic Acid" OR " trans-Retinoic Acid " OR "trans Retinoic Acid" OR " Retin A" OR " Alitretinoin" OR " 9-cis-Retinoic Acid" OR " cis Retinoic Acid" OR "Vitamin E" OR "Tocopherol*" OR "Tocovital" OR "VitaE" OR "Uno Vit" OR "alpha-Tocopherol" OR "alpha Tocopherol" OR "beta-Tocopherol" OR "gamma-Tocopherol" OR "Omega-3 " OR " Omega 3" OR " Omega3 " OR "n-3 Oil " OR "n 3 PUFA" OR " n3 Fatty Acid " OR "n3 Polyunsaturated Fatty Acid" OR " n3 Oil*" OR " n 3 Polyunsaturated Fatty Acid" OR "alpha Linolenic Acid" OR "alpha-Linolenic Acid" OR "Linolenic Acid" OR "Docosahexenoic Acid*" OR "Docosahexaenoate" OR "Eicosapentaenoic Acid" OR "Dietary Supplement* " OR "Supplements Dietary " OR "Supplement*" OR "Nutraceutical* " OR "Fish Oil* " OR "Fish Liver Oil*" OR "Ascorbic Acid" OR "Vitamin C" OR "Ascorbate" OR "Vitamin B 6" OR "Pyridoxal" OR "Pyridoxin" OR "Pyridoxol" OR "Vitamin B 6" OR "Vitamin B6" OR "Vitamin B 2" OR "Vitamin B2" OR "Vitamin B 12" OR "Vitamin B12" OR "Vitamin B" OR "Riboflavin" OR "Riboflavin" OR "Vitamin B 12" OR "Cobalamins" OR "Cobalamin" |
|  | final | 1 AND 2 |
| WebOF sience | 1 | ALL=(Aortic Aneurysm OR Abdominal Aortic Aneurysm OR Abdominal Aorta Aneurysm OR Thoracoabdominal Aortic Aneurysm) |
|  | 2 | ALL=( Vitamin D3 OR Vitamin D OR Cholecalciferol OR Hydroxycholecalciferol OR Hydroxyvitamins D OR 25-Hydroxyvitamin D OR 25 Hydroxyvitamin D OR 25-Hydroxycholecalciferol OR 25 Hydroxycholecalciferol OR Dihydroxycholecalciferol OR Dihydroxyvitamins D OR Calcitriol OR 1,25-Dihydroxycholecalciferol OR 1,25 Dihydroxycholecalciferol OR 1,25-Dihydroxyvitamin D3 OR 1,25 Dihydroxyvitamin D3) |
|  | 3 | ALL= (Vitamin A OR Retinol OR All Trans Retinol OR Tretinoin OR Retinoic OR all-trans-Retinoic Acid OR Retinoic Acid OR Retin A OR Alitretinoin OR 9-cis-Retinoic Acid OR Vitamin E OR Tocopherol OR alpha-Tocopherol OR alpha Tocopherol OR beta-Tocopherol OR gamma-Tocopherol) |
|  | 4 | ALL=(Omega-3 OR Omega 3 OR omegad OR n-3 Oil OR n 3 PUFA OR n3 Fatty Acid OR n3 Polyunsaturated Fatty Acid OR n3 Oil OR n 3 Polyunsaturated Fatty Acid OR Linolenic Acid OR docosahexaenoic OR Docosahexaenoate OR Eicosapentaenoic Acid OR Supplement OR Nutraceutical OR Fish Liver Oil) |
|  | 5 | ALL= (Ascorbic Acid OR Vitamin C OR Ascorbate OR Vitamin B 6 OR Pyridoxal OR Pyridoxin OR Pyridoxol OR Vitamin B 6 OR Vitamin B6 OR Vitamin B 2 OR Vitamin B2 OR Vitamin B 12 OR Vitamin B12 OR Vitamin B OR Riboflavin OR Riboflavin OR Vitamin B 12 OR Cobalamins OR Cobalamin) |
|  | Final | (#1) AND ( #2 OR #3 OR #4) |
